# Reduced recognition of facial emotional expressions in global burnout and burnout depersonalization in healthcare providers

**DOI:** 10.7717/peerj.10610

**Published:** 2021-01-13

**Authors:** Valentina Colonnello, Luca Carnevali, Paolo Maria Russo, Cristina Ottaviani, Valeria Cremonini, Emanuele Venturi, Katia Mattarozzi

**Affiliations:** 1 Department of Experimental, Diagnostic and Specialty Medicine, University of Bologna, Bologna, Italy; 2 Stress Physiology Lab, Department of Chemistry, Life Sciences and Environmental Sustainability, University of Parma, Parma, Italy; 3 Department of Psychology, Sapienza University of Rome, Rome, Italy; 4 Neuroimaging Laboratory, IRCCS Santa Lucia Foundation, Rome, Italy; 5 IRCCS Institute of Neurological Sciences of Bologna, Bologna, Italy

**Keywords:** Emotion recognition, Healthcare providers, Burnout, Experimental task, Nurses

## Abstract

The healthcare provider profession strongly relies on the ability to care for others’ emotional experiences. To what extent burnout may relate to an actual alteration of this key professional ability has been little investigated. In an experimentally controlled setting, we investigated whether subjective experiences of global burnout or burnout depersonalization (the interpersonal component of burnout) relate to objectively measured alterations in emotion recognition and to what extent such alterations are emotion specific. Healthcare workers (*n* = 90) completed the Maslach Burnout Inventory and a dynamic emotion recognition task in which faces with neutral emotional expressions gradually changed to display a specific basic emotion (happiness, anger, fear, or sadness). Participants were asked to identify and then classify each displayed emotion. Before the task, a subsample of 46 participants underwent two salivary cortisol assessments. Individuals with global burnout were less accurate at recognizing others’ emotional expressions of anger and fear, tending to misclassify these as happiness, compared to individuals without global burnout. Individuals with high burnout depersonalization were more accurate in recognizing happiness and less accurate in recognizing all negative emotions, with a tendency to misclassify the latter as positive ones, compared to healthcare workers with moderate/low depersonalization. Moreover, individuals with high depersonalization—but not participants with global burnout—were characterized by higher cortisol levels. These results suggest that the subjective burnout experience relates to an actual, but selective, reduction in the recognition of facial emotional expressions, characterized by a tendency to misclassify negative emotional expressions as positive ones, perhaps due to an enhanced seeking of positive social cues. This study adds to the understanding of emotional processing in burnout and paves the way for more nuanced studies on the role of altered processing of threat signals in the development and/or persistence of burnout.

## Background

The ability to recognize others’ emotions plays a key role in the healthcare context, where patients share emotional aspects of their lives (Colonnello et al., 2020; Levinson, Gorawara-Bhat & Lamb, 2000) and healthcare providers are called to respond to patients’ emotions (Epstein et al., 2007; Larson & Yao, 2005). The focus of the present study is to investigate the extent to which the ability to recognize emotional cues may be objectively challenged in healthcare providers experiencing burnout.

According to Maslach (2017), Maslach et al. (1986) and Maslach & Jackson (1981), burnout is a multifaceted phenomenon characterized by emotional exhaustion (depletion of personal resources), depersonalization (perception of excessive detachment), and reduced personal accomplishment (suffering from reduced efficacy at work).

Several studies document the social relevance of experiencing burnout (Serenari et al., 2019; Chirico et al., 2019; Adriaenssens, De Gucht & Maes, 2015; Aiken et al., 2012; Salyers et al., 2017) and the relationship between the subjective experience of burnout and actual clinical outcomes (Taris, 2006). For example, burnout depersonalization has been found to be associated with suboptimal patient care (Shanafelt et al., 2002), longer recovery times for patients post discharge (Halbesleben & Rathert, 2008), and increased ombudsman complaints (Windover et al., 2018).

A first step in counteracting the negative social effects of burnout might be elucidating possible alterations of social stimulus processing in individuals experiencing burnout. Specifically, questions arise regarding whether subjective burnout and high depersonalization experience relate to an objective alteration in the ability to recognize others’ emotions. Given the need for evidence-based research on burnout in healthcare providers (Chirico & Magnavita, 2020; Low et al., 2019) and the high risk of burnout in nurses (Maslach, 2003; Clegg, 2001; McVicar, 2003), the nursing population is one of the most appropriate for addressing these questions.

Previous experimental evidence from non-healthcare professions indicates that the processing of emotional cues is altered in individuals with burnout. For example, Sokka et al. (2014) report that individuals with burnout show a faster attention capture tendency for emotionally negative over positive auditory stimuli. In addition, Bianchi et al. (2018a) suggest that individuals with burnout show an increased memory recall for negative over positive words.

Here, we hypothesize that subjective burnout relates to an actual alteration in the recognition of salient social stimuli, that is, facial emotional expressions. On the basis of data linking subjective burnout with reduced patient satisfaction (Windover et al., 2018), we expect that healthcare workers with burnout will show actual difficulties in recognizing others’ emotions. In addition, previous studies indicating that individuals with burnout show alterations in the processing of negative emotional stimuli (Sokka et al., 2014; Bianchi et al., 2018a) lead us to expect that individuals with burnout will show an emotion-specific alteration in recognizing others’ negative emotions. Finally, given that among the three burnout dimensions, depersonalization plays the most important role in the quality of interpersonal interactions (Maslach, 2017; Maslach et al., 1986; Maslach & Jackson, 1981) and affects healthcare outcomes (Shanafelt et al., 2002), our major aim is to examine the distinctive role of both global burnout and depersonalization experience on emotion recognition ability.

In the past decade, cortisol, a golden biomarker of stress-related hypothalamus pituitary adrenal (HPA) axis regulation (Kirschbaum & Hellhammer, 1989), has been proposed as a biomarker or even a predictor of burnout. For example, Marchand et al. (2014) reported that levels of burnout symptoms were steadily associated with lower cortisol concentrations from 14:00 h until bedtime (Marchand et al., 2014). However, results have not always been consistent (e.g., Wingenfeld et al., 2009; Grossi et al., 2005; Mommersteeg et al., 2006), likely due to the fact that different burnout symptom subtypes appear to be associated with different cortisol profiles (Marchand et al., 2014). To the best of our knowledge, though, burnout depersonalization has never been considered in association with cortisol. The present study aims to fill this gap by examining the association between daily cortisol and both global burnout and symptoms of depersonalization. This sub-aim is strictly related to our major aim, as it has been shown that increases in cortisol which are induced by psychosocial stressors are associated with increased selective attention to threatening faces and enhanced processing of angry faces (Dandereau et al., 2007; Wieser et al., 2010).

## Methods

### Participants

From October 2017 through January 2018, a total of 90 nurses (9 men, 81 women; age: *M* = 45.06; SD = 9.45 years; work experience: *M* = 21.88, SD = 10.73 years, all Caucasians) working at the local university hospital were recruited by two trained undergraduate interviewers. The eligibility criteria were having at least 3 months’ work experience, correct or corrected-to-normal vision, and no history of neurological or psychiatric diseases and being native Italian speakers.

Within 1 week after the emotion recognition task (see below), participants completed the Italian version of the Maslach Burnout Inventory (MBI, Sirigatti, 1993). According to the recommended cutoff for defining burnout among Italian healthcare providers (Sirigatti, 1993), participants were considered as belonging to the burnout group if they had both a high emotional exhaustion score (≥24) and a high depersonalization score (≥9); remaining participants were regarded as belonging to the non-burnout group.

In a randomly selected subsample of participants (*n* = 46), saliva samples were collected to measure salivary cortisol (Hellhammer, Wüst & Kudielka, 2009). These participants were instructed not to smoke, eat, or drink for 30 min prior to the assessment. Two saliva samples were collected 7 min apart at the end of participants’ shifts (i.e., 14:00 h, *n* = 21; 20:00 h, *n* = 25), immediately prior to the emotion recognition task. Samples were collected using oral swabs and swab storage tubes (Sarstedt, Rommelsdorf, Germany) and stored at −20 °C from the completion of the session until biochemical analysis. At that time, samples were thawed, brought to room temperature and centrifuged (1,500*g*× 10 min), resulting in a clear supernatant of low viscosity. Cortisol levels were assayed in duplicates following kit instructions with a 96-well plate enzyme-linked immunosorbent assay (High Sensitivity Salivary Cortisol Enzyme Immunoassay Kit: Salimetrics LLC, State College, PA, USA), using the Infinite F50 plate reader and Magellan software (TecanGroup Ltd, Männedorf, Switzerland).

All participants signed informed consent prior to the study and were fully debriefed at its conclusion. The experimental procedures were approved by the University of Bologna Institutional Review Board (IRB; approval number: CE 17068).

### Emotion recognition task

For the task, we used the same procedure and stimuli reported in Colonnello, Mattarozzi & Russo (2019) and in Colonnello, Russo & Mattarozzi (2019). Participants viewed a total of 72 video clips (10 s each, frame rate: 25 frames/s) showing a neutral facial expression gradually and continuously changing from 0% to 100% into a basic full-intensity facial emotional expression (happiness, anger, fear, or sadness).

The video stimuli were constructed using 18 neutral and full emotional expressions (happiness, anger, fear, sadness) of 18 Caucasian actors from the Karolinska Directed Faces Database (Lundqvist & Litton, 1998). For the practice trials, we used two additional videos presenting the morphing from neutral into full emotional expressions of two faces from the Karolinska Directed Faces Database.

Participants were instructed to view each video and stop it as soon as they felt certain that they identified a specific emotion. Immediately after that, participants viewed the stopped frame on the center screen and selected, in a forced-choice task, the label (happiness, anger, fear, or sadness) that best matched the displayed emotion. There was no time limit or feedback throughout the task. We used E-Prime software (http://www.pstnet.com/) for stimulus presentation and response data collection.

### Statistical analysis

Differences between burnout groups were assessed using the unpaired Student’s *t* test for continuous variables (age and work experience). For the emotion recognition task, the accuracy (percentage of emotions correctly recognized) and speed (milliseconds needed to correctly identify the emotion) were analyzed using two separate multivariate ANOVAs, with either global Burnout (burnout vs. non-burnout) or Depersonalization (high vs. low) treated as a between-subjects factor. The specificity of emotion alteration and misclassification was tested using univariate tests. For the emotional expressions recognized least, we calculated the percentage of times another emotion was misclassified (e.g., the percentage of times anger and fear expressions were incorrectly labeled as happiness or sadness for global burnout).

In second-step analyses, we introduced relevant covariates (age and work experience) into the model; however, because no significant main effects or interactions of these factors were found (all ps > 0.05), the analysis was repeated excluding these factors from the statistical models.

Regarding cortisol, the role of potential confounders (i.e., age, experience, and time of assessment) was first examined by *t* tests and Pearson’s correlations. Among the examined variables, only those significantly associated with cortisol values (i.e., average of the two samples) were included in a univariate ANOVA with global Burnout (burnout vs. without-burnout) and Depersonalization (high vs. low) as the between-subjects factors and average cortisol values as the dependent variable.

## Results

**Figure 1:**
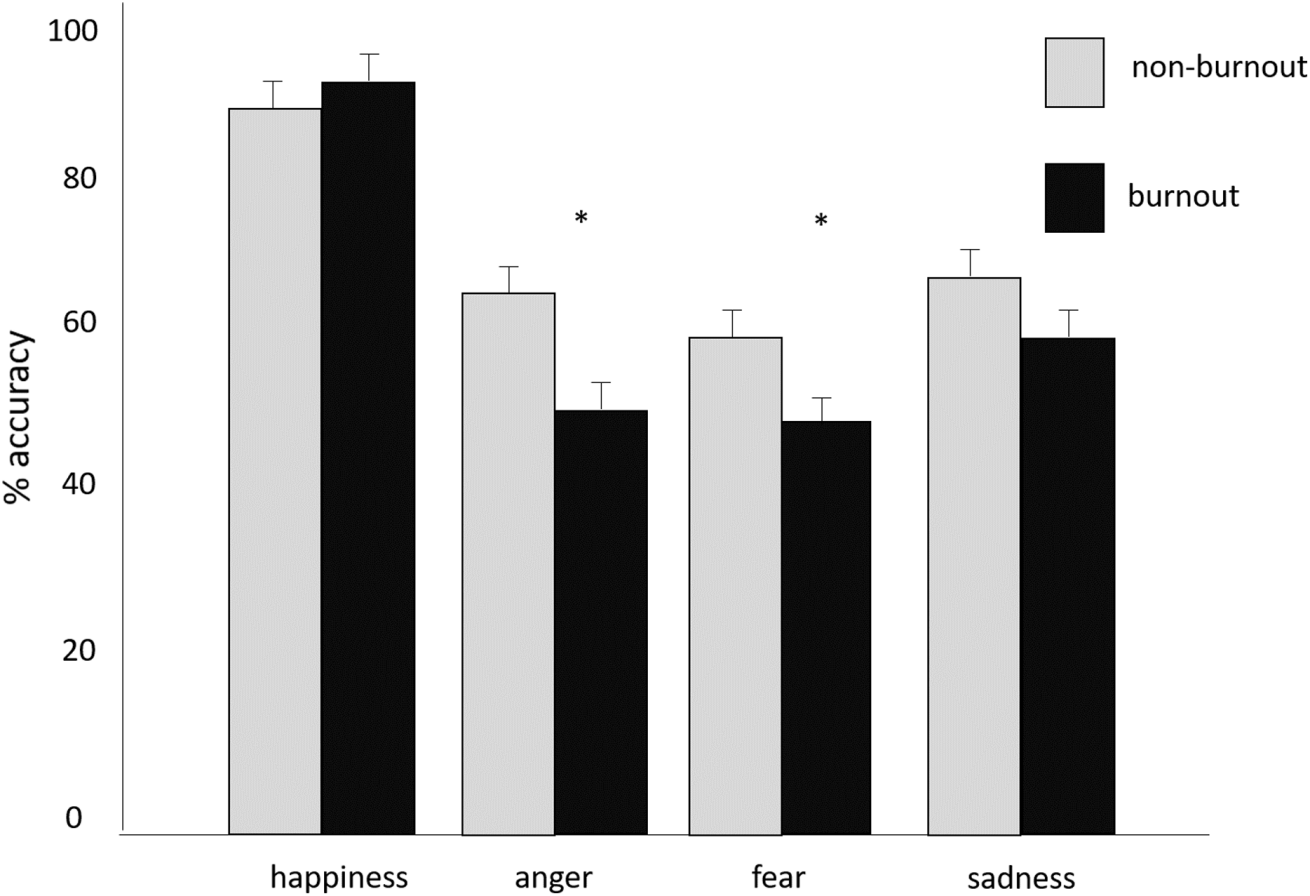
Emotion Recognition Accuracy and Speed. Mean + SE of percentage of emotions correctly recognized by burnout and non-burnout groups. *p < 0.05.

Of the 90 participants, 26 reported experiencing global burnout. The two global burnout groups did not differ for age, *t*(88) = 1.27, *p* > 0.05, and work experience, *t*(88) = 1.28, *p* > 0.05. On the emotion recognition task, the burnout group was less accurate than the non-burnout group at recognizing others’ emotions, *F*(4, 85) = 5.35; *p* = 0.001; η_*p*_^*2*^ = 0.20. Univariate differences showed the burnout group tended to recognize expressions of anger, *F*(1,88) = 8.52; *p* = 0.004, η_*p*_^*2*^ = 0.06, and fear, *F*(1,88) = 5.51; *p* = 0.02, η_*p*_^*2*^ = 0.09, less accurately. The emotion recognition accuracy for the specific emotions is depicted in Fig. 1. In addition, the burnout group tended to misclassify anger and fear as happiness, *F*(1,88) = 8.73; *p* = 0.004; η_*p*_^*2*^ = 0.09, but not as sadness (*p* > 0.05).

Regarding the depersonalization experience, 45 participants reported highly frequent depersonalization (high depersonalization group), while the remaining 45 reported moderate/low depersonalization scores (low depersonalization group). Nurses with the most frequent depersonalization experience were less able to recognize others’ emotions, *F*(4, 85) = 9.64; *p* < 0.0001; η_*p*_^*2*^ = 0.31. Univariate analysis revealed that individuals with high depersonalization experience tended to less accurately recognize all negative emotions: anger, *F*(1,88) = 5.35; *p* = 0.02, η_*p*_^*2*^ = 0.06; fear, *F*(1,88) = 4.01; *p* = 0.049, η_*p*_^*2*^ = 0.04; and sadness, *F*(1,88) = 4.08; *p* = 0.046, η_*p*_^*2*^ = 0.04.

Happiness was recognized better by individuals with high depersonalization scores than by individuals with low depersonalization scores, *F*(1,88) = 5.84; *p* = 0.020, η_*p*_^*2*^ = 0.06 (S1). Consistent with the global burnout results, individuals with highly frequent depersonalization experience tended to misclassify negative emotions as positive ones, *F*(1,88) = 5.82; *p* = 0.018; η_*p*_^*2*^ = 0.06.

Emotion recognition speed was not affected by either global burnout or burnout depersonalization (ps > 0.05).

Preliminary analyses of cortisol levels found a significant association with the time of assessment (early afternoon vs. late afternoon, *t*(44) = 2.49, *p* = 0.02), with lower cortisol levels in participants enrolled during the late afternoon (*n* = 25) compared to the early-afternoon shift (*n* = 21). No differences due to age and work experience emerged; therefore, only the time of assessment was included in the univariate ANOVA as a factor.

The model yielded a significant main effect of depersonalization, *F*(1,40) = 5.29; *p* = 0.03, η_*p*_^*2*^ = 0.12, and a marginally significant effect of time of assessment, *F*(1,40) = 3.68; *p* = 0.06, η_*p*_^*2*^ = 0.08. No significant effect of global burnout or interactions emerged. Pairwise comparisons showed higher cortisol levels in the high-depersonalization group compared to the low-depersonalization group (*p* = 0.04; 0.13 ± 0.02 vs. 0.08 ± 0.02, respectively).

## Discussion

Though it has not been classified as a medical condition, burnout syndrome is a widely reported occupational disease risk among healthcare professionals (Chirico, 2017) that deserves attention from scholars and physicians (Chirico & Magnavita, 2020; Chirico et al., 2019). Thus, the overarching aim of the present study was to shed light on the relationship between subjective burnout experience and objective alteration of emotion recognition, a central component in healthcare interactions. In a sample of nurses presenting burnout with a prevalence similar to that observed in larger samples of healthcare providers (Poncet et al., 2007), we found that the subjective burnout experience is characterized by a selective difficulty in recognizing others’ emotions. Resonating with and extending previous findings documenting alterations in the processing of emotionally negative stimuli due to burnout (Sokka et al., 2014; Bianchi et al., 2018a), we found differences between the burnout and non-burnout groups related to the recognition of negative emotional expressions. Specifically, individuals with burnout recognized the negative emotional expressions of anger and fear less accurately. Our inspection of emotion misclassification revealed an intriguing pattern: compared to those without burnout, individuals with burnout were more likely to mislabel negative emotional expressions of anger and fear as positive ones.

As documented elsewhere, facial expressions of anger and fear are social cues signaling potential threat (Whalen et al., 2001; Zald, 2003), while facial expressions of happiness are regarded as essential social reward cues (Monk et al., 2008). Thus, individuals with burnout specifically tended to recognize social signals of threats less accurately and misclassify them as rewarding signals.

Interestingly, depersonalization experience, the core interpersonal dimension of burnout, relates to a specific difficulty in recognizing all negative emotions, a potentiated ability to accurately recognize positive emotions, and a tendency to misread negative emotions as positive emotions. Thus, compared to those from global burnout, difficulties from depersonalization extend to sadness expressions, a social cue that may elicit caring behavior (Seidel et al., 2010). In addition, individuals who experience high depersonalization had elevated basal cortisol levels.

Taken together, the present results may reflect that burned-out healthcare providers have a lack of sensitivity to threat signals in conjunction with a seeking for positive, rewarding social signals. Thus, it is possible that healthcare providers experiencing burnout scan the available social cues for social incentives at the cost of misreading others’ threat cues. This finding apparently contrasts with previous findings suggesting that individuals with burnout tend to provide a negative interpretation of ambiguous scenarios (Bianchi et al., 2018b). However, methodological differences between populations (healthcare providers vs. heterogeneous work population) and procedures (i.e., classifying facial expressions vs. imagining specific complex scenarios) might have contributed to such differences.

This result also apparently conflicts with the idea that cortisol alterations may mediate the association between burnout and impairments in emotion recognition. Whereas cortisol increases have been associated with enhanced sensitivity to negative emotions (Dandereau et al., 2007; Wieser et al., 2010), our study found that participants with high-depersonalization symptoms had higher cortisol levels but also a tendency to misrecognize negative emotions as positive. Unfortunately, the non-longitudinal nature of our data prevents us from examining plausible mediation models, but at a purely speculative level, it is possible that the high cortisol-high depersonalization-positive bias we found is somehow related to the role of glucocorticoids in the reward system (Putman et al., 2010). It has to be noted, however, that in this study, only depersonalization symptoms and not overall burnout were associated with significant cortisol changes, while impaired emotion recognition was present in both categories, suggesting that cortisol may not be involved in such impairments (as also suggested by Duesenberg et al., 2016).

Unfortunately, as already mentioned, we cannot trace the temporal causal relation between burnout experience and emotion recognition alteration. Thus, we cannot rule out whether the burned-out individuals became less able to recognize emotions or whether they have always been less effective at recognizing and classifying others’ emotional expressions. An additional limitation of this study is the relatively small sample size, which suggests caution in generalizing these conclusions. Further, the participants were mainly women. Even though this sex ratio parallels the ratio observed in national samples of nurses, it is not possible to advance conclusions on the relationship between sex and burnout experience and, therefore, emotion recognition alteration.

To conclude, this study provides the first glimmers of the relationship between healthcare providers’ global burnout and depersonalization experience and their alteration of facial emotional expression processing. Our findings suggest investigating this topic further, focusing on the processing of threat signals and reward-seeking motivation in healthcare providers prone to experiencing burnout. The results also have implications for further refining of burnout measurements. For example, the MBI items do not explore whether individuals with burnout are aware of their tendency to misrecognize different affective states. Such measurements may aid understanding of the burnout depersonalization experience and perhaps reveal the course of burnout manifestation and its role in progressively jeopardizing the quality of interpersonal interactions.

## Supplemental Information

10.7717/peerj.10610/supp-1 Supplemental Information 1 Emotion recognition accuracy and speed for each participant.

10.7717/peerj.10610/supp-2 Supplemental Information 2 Mean (M) and Standard Deviation (SD) of emotion recognition accuracy (% of correct responses).

## Additional Information and Declarations

### Data Availability

The following information was supplied regarding data availability: The raw measurements are available in the Supplemental Files.
