# Supplementary material for: Reduced recognition of facial emotional expressions in global burnout and burnout depersonalization in healthcare providers
Source: PeerJ. 2021 Jan 13;9:e10610. doi: 10.7717/peerj.10610 (PMC7811292; doi:10.7717/peerj.10610)
Supplement: Supplemental Information 2 [file peerj-09-10610-s002.docx]

**S1.**

**Table 1.** Mean (M) and Standard Deviation (SD) of emotion recognition accuracy (% of correct responses).

|  |  | **Burnout** | |  | | **Non burnout** | | |  | |  |  |  |  |
| --- | --- | --- | --- | --- | --- | --- | --- | --- | --- | --- | --- | --- | --- | --- |
|  |  | M | SD |  | | M | | SD |  | |  |  |  |  |
|  |  |  |  |  | |  | |  |  | |  |  |  |  |
|  | Happiness | 91.24 | 19.19 |  | | 86.8 | | 21.26 |  | | *F*(1,88) = 0.85; *p* = .360, *η_p_^2^*  = .01 | | | |
|  | Anger | 50.85 | 23.13 |  | | 65.19 | | 20.27 |  | | *F*(1,88) = 8.52; *p* = .004, *η_p_^2^*  = .06 | | | |
|  | Fear | 49.57 | 15.23 |  | | 60.33 | | 21.23 |  | | *F*(1,88) = 5.51; *p* = .021, *η_p_^2^*  = .09 | | | |
|  | Sadness | 59.19 | 16.99 |  | | 67.01 | | 23.69 |  | | *F*(1,88) = 2.34; *p* = .130, *η_p_^2^*  = .03 | | | |
|  |  |  |  |  | |  | |  |  | |  |  |  |  |
|  |  | **High depersonalization** | | |  | | **Low depersonalization** | | |  |  |  |  |  |
|  |  | M | SD |  | | M | | SD |  | |  |  |  |  |
|  |  |  |  |  | |  | |  |  | |  |  |  |  |
|  | Happiness | 93.21 | 15.07 |  | | 82.96 | | 24.17 |  | | *F*(1,88) = 5.84; *p* = .020, *η_p_^2^*  = .06 | | | |
|  | Anger | 55.80 | 20.54 |  | | 66.30 | | 22.36 |  | | F(1,88) = 5.35; *p* = .020, *η_p_^2^*  = .06 | | | |
|  | Fear | 52.96 | 16.78 |  | | 61.48 | | 22.52 |  | | *F*(1,88) = 4.01; *p* = .049, *η_p_^2^*  = .04 | | | |
|  | Sadness | 60.12 | 17.78 |  | | 69.38 | | 25.17 |  | | *F*(1,88) = 4.08; *p* = .046, *η_p_^2^*  = .04 | | | |
|  |  |  |  |  | |  | |  |  | |  |  |  |  |
